# Supplementary material for: De novo protein structure prediction using ultra-fast molecular dynamics simulation
Source: PLoS One. 2018 Nov 20;13(11):e0205819. doi: 10.1371/journal.pone.0205819 (PMC6245515; doi:10.1371/journal.pone.0205819)
Supplement: S1 Text — (PDF) [file pone.0205819.s001.pdf]

Supplemental Text

Methods

Network structure of *Phsior*

The parameters of *Phsior* were achieved by being trained on a set of proteins consisting of 8,000 chains and it was tested using 2,586 chains. *Phsior* consists of seven layers (as illustrated in Fig 2), which are set to use in this study. The first layer is a convolutional layer containing sixteen feature maps, each of which has a kernel of 5×5. In the input to each first layer neuron, features are filtered by the kernel. Then, it is followed by a max-pooling layer to pick up larger-scale detail, which outputs the maximum value in each 3×3 pixel square from the previous outputs. Again, another convolutional layer consisting of 32 kernels of 1×1 is the third layer, followed by a same size convolutional layers as previous one with 48 feature maps. The sixth and seventh layers are the fully connected layers to generate 4 neurons producing the final outputs. Finally, a *TANH* activation function is applied to the neuron output to filter the results.

Training and test of *Phsior*

In the training phase, we initialized the all kernels weights in *Phsior* using a uniform distribution of near-zero values. Log squared residual ($\log((y - y')^2)$) between the *Phsior* output and the real-values of torsion angles (ϕ, ψ) is used as the loss function. To optimize the parameters, we use stochastic gradient descent with a batch size of 32. By default, the neural network is trained for 200 echoes. The learning rate is set to 0.01 in the first iteration and decreased by 0.005 after each iteration. In all the convolutional layers, we set the stride of the filters to a default value, that is 1. Since the size of the proposed *Phsior* is not so large, dropout was not used in building the predictor. To train the network, Nesterov Accelerated Gradient algorithm [1] was employed to find the parameters of the network for the given training and test data sets. *Phsior* is trained using 35×24 pixel matrix extracted from protein sequences for each amino acid. The features in the matrix include PSSM, SS, and SA, which are the data used to train the CNN in *Phsior*. On the other hand, we normalized the data in the test protein chains and launched the *Phsior* to validate its performance. The predictive performance of the *Phsior* was demonstrated on the eighteen proteins as show in Fig 3 and S2 Fig.

Constraint of torsion angels

Since the *Upside* runs the simulations dependent on the Ramachandran map distribution [2, 3], in this study, we applied the predicted torsion angles (ϕ, ψ) as constraints to launch the *Upside* simulations from an extended structure. For the ith residue, we defined a range for the torsional angle ϕ_i as follows,

$$\phi_i \in [\phi_i^{pred} - 20°, \phi_i^{pred} + 20°]. \quad \text{(A)}$$

Similarly, the range of ψ_i is obtained as follows,

$$\psi \in [\psi_i^{pred} - 20°, \psi_i^{pred} + 20°]. \quad \text{(B)}$$

Potential for contacted residues

The residue-contacts were calculated using the Eq. (3), and then we convert the probabilities of contact between pairwise residues to an energy potential as follows,

$$E = \frac{E_0}{1 + e^{w_0(r-r_0)}}, \quad \text{(C)}$$

where r and r_0 are distance of pairwise C_α atoms and threshold, respectively. E_0 is the score computed from plmDCA. The score E_0 is illustrated in Fig 4 and S3 Fig. w_0 is the width of the potential, while E_0 is initial energy of residue contacts converted from the strength between the pairwise residues.

Structure clustering

Using the proposed protocol, for each protein sequence, the *Upside* was launched 500 simulations to generate a total of 25,000 models. Among the top 50 models generated by the *Upside* for each simulation trajectory, there will be many structures of similar folds. Moreover, two models of similar folds can also be found in two different trajectories. Therefore, it is necessary to classify the structures into a much smaller number of distinct clusters. In this study, we used *fast_protein_cluster* software [4] to cluster the structures into five groups, and the centroid model in the largest cluster were selected as our ”blind predicted models” as shown in Fig 7 and S4 Fig.

Computational time and comparison

In the developed NiDelta, we launched the molecular dynamics (MD) simulation on each benchmark protein on computer clusters, in which each node is configured with 20 Intel(R) Xeon(R) CPU E5-2650 v3 @ 2.30GHz, and the computational time is illustrated in S1 Fig. For example, the running time on the largest protein (MBP, PDB ID: 1DMB) of 370 residues is 43917.3 CPU seconds, about 12.2 CPU hours. Accordingly, the developed approach is much faster than the classical MD-based methods for protein structure prediction.

References

[1] Ilya Sutskever, James Martens, George Dahl, and Geoffrey Hinton. On the importance of initialization and momentum in deep learning. In *International conference on machine learning*, pages 1139–1147, 2013.

[2] John M Jumper, Karl F Freed, and Tobin R Sosnick. Maximum-likelihood, self-consistent side chain free energies with applications to protein molecular dynamics. *arXiv preprint arXiv:1610.07277*, 2016.

[3] John M. Jumper, Karl F. Freed, and Tobin R. Sosnick. Trajectory-based parameterization of a coarse-grained forcefield for high-throughput protein simulation. *bioRxiv*, 2017.

[4] Ling-Hong Hung and Ram Samudrala. fast_protein_cluster: parallel and optimized clustering of large-scale protein modeling data. *Bioinformatics*, 30(12):1774–1776, 2014.

[5] David T Jones, Tanya Singh, Tomasz Kosciolek, and Stuart Tetchner. Metapsicov: combining coevolution methods for accurate prediction of contacts and long range hydrogen bonding in proteins. *Bioinformatics*, 31(7):999–1006, 2014.

[6] Baoji He, SM Mortuza, Yanting Wang, Hong-Bin Shen, and Yang Zhang. Nebcon: protein contact map prediction using neural network training coupled with naïve bayes classifiers. *Bioinformatics*, 33(15):2296–2306, 2017.
