# Supplementary figures and images for: De novo protein structure prediction using ultra-fast molecular dynamics simulation
Source: PLoS One. 2018 Nov 20;13(11):e0205819. doi: 10.1371/journal.pone.0205819 (PMC6245515; doi:10.1371/journal.pone.0205819)

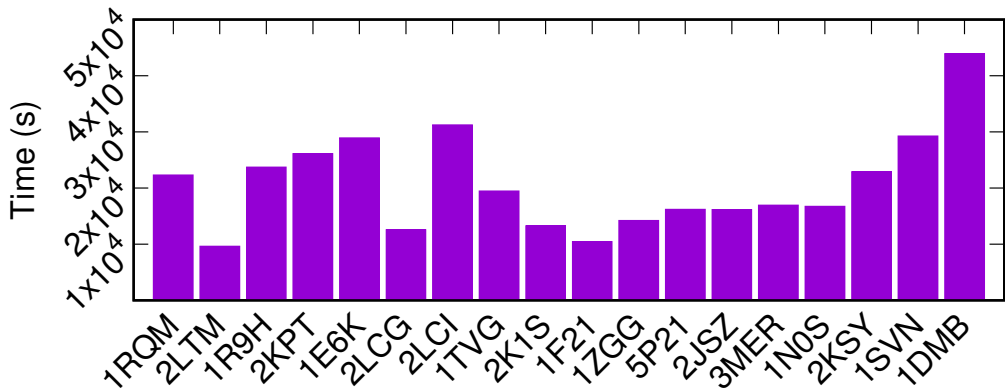

Figure S1: Computational time on each protein.

Supplement: S1 Fig — (PDF) [file pone.0205819.s002.pdf]

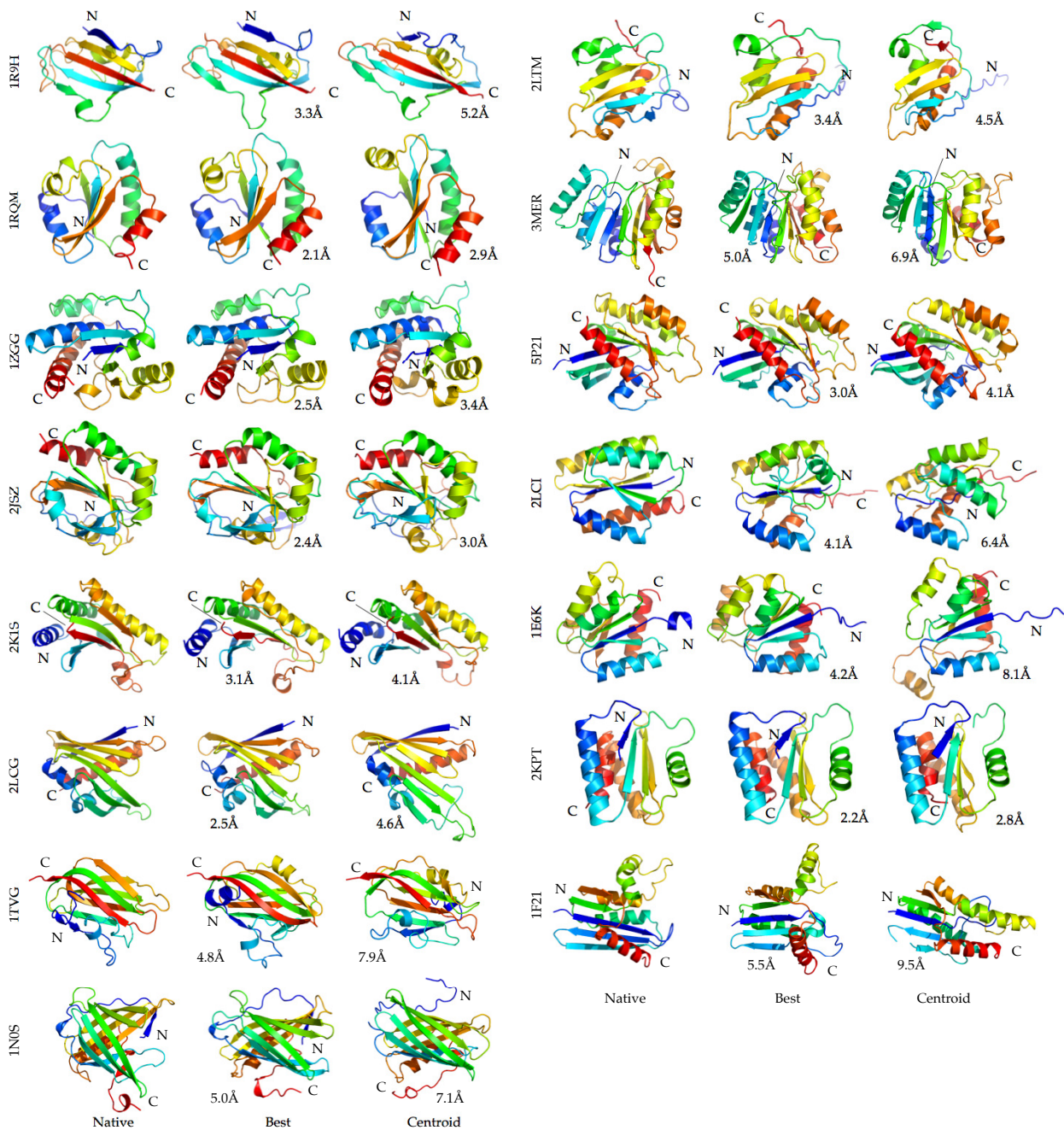

Figure S4: Visual comparisons on the highlighted predicted models of the target proteins.

Supplement: S4 Fig — (PDF) [file pone.0205819.s005.pdf]
