# Supplementary material for: De novo protein structure prediction using ultra-fast molecular dynamics simulation
Source: PLoS One. 2018 Nov 20;13(11):e0205819. doi: 10.1371/journal.pone.0205819 (PMC6245515; doi:10.1371/journal.pone.0205819)
Supplement: S2 Fig — (a) MAE comparison of ϕ, and (b) MAE comparison of ψ. (PDF) [file pone.0205819.s003.pdf]

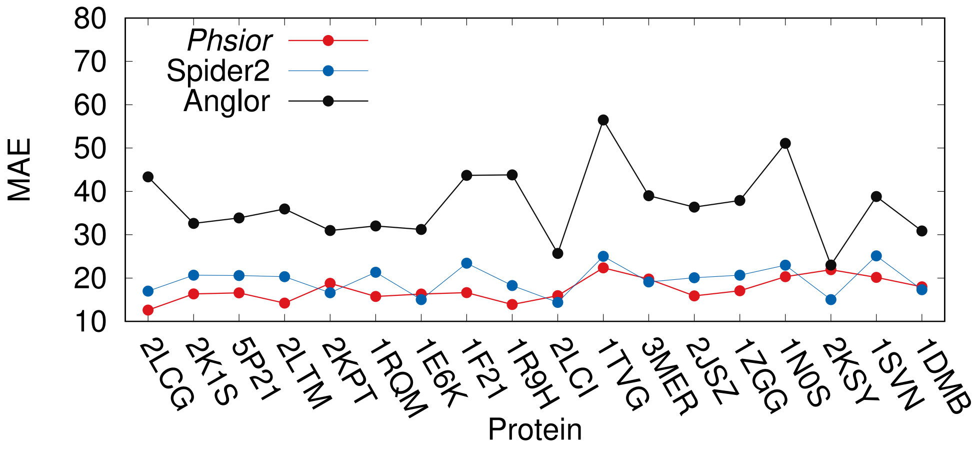

(a)

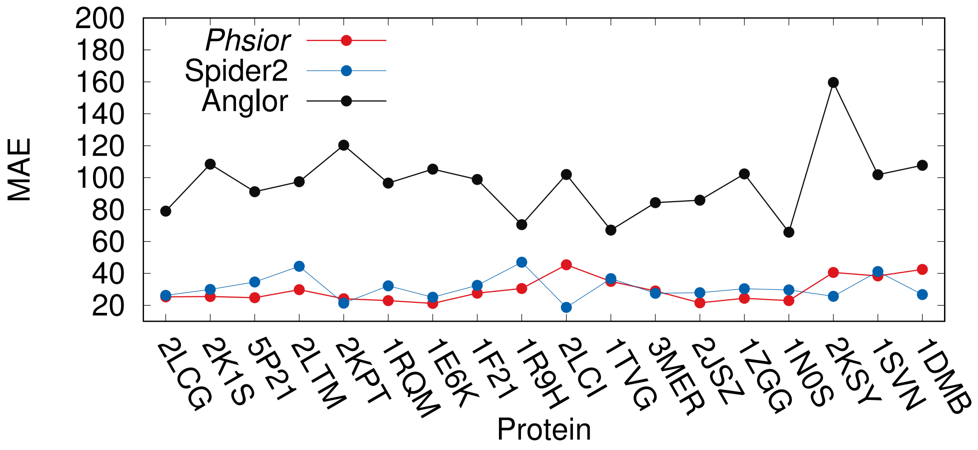

(b)

Figure S2: Comparison on the MAE of the predicted torsion angles (ϕ, ψ) among Anglor, Spider2, and *Phsior*. (a) MAE comparison of ϕ, and (b) MAE comparison of ψ.
