# Supplementary material for: De novo protein structure prediction using ultra-fast molecular dynamics simulation
Source: PLoS One. 2018 Nov 20;13(11):e0205819. doi: 10.1371/journal.pone.0205819 (PMC6245515; doi:10.1371/journal.pone.0205819)
Supplement: S3 Fig — All the residue-contacts (top 2L) used in the Upside simulations are shown in blue filled squares. The native and estimated residue-contacts are in red and blue, respectively. The dots in green circles are noises (false positive inferences). (PDF) [file pone.0205819.s004.pdf]

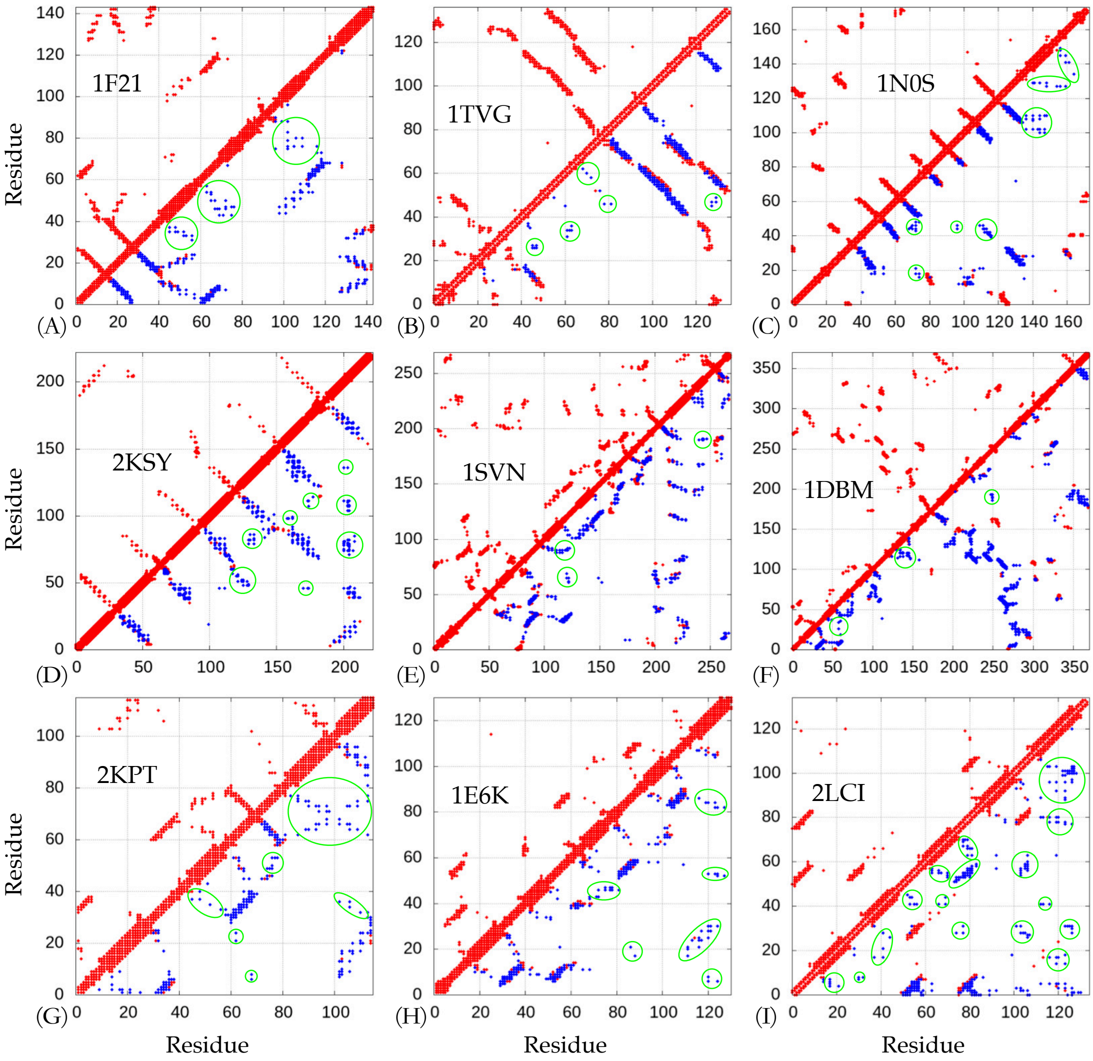

Figure S3: The predicted residue-contacts for highlighted targets listed Table 1. All the residue-contacts (top $2L$) used in the *Upside* simulations are shown in blue filled squares. The native and estimated residue-contacts are in red and blue, respectively. The dots in green circles are noises (false positive inferences).
