# Supplementary material for: De novo protein structure prediction using ultra-fast molecular dynamics simulation
Source: PLoS One. 2018 Nov 20;13(11):e0205819. doi: 10.1371/journal.pone.0205819 (PMC6245515; doi:10.1371/journal.pone.0205819)
Supplement: S1 Table — (PDF) [file pone.0205819.s006.pdf]

Table S1: The key layers in *Phsior* with convolutional and fully connected layers.

Layer	Specification	Specification
Input	35x24x3	–
Convolution	5x5x3x16	ReLU
Max-Pooling	3x3x3	Strides=2
Convolution	1x1x3x32	ReLU
Convolution	5x5x3x48	ReLU
Max-Pooling	2x2x3	Strides=2
Softmax	4	Tanh
