# Supplementary material for: De novo protein structure prediction using ultra-fast molecular dynamics simulation
Source: PLoS One. 2018 Nov 20;13(11):e0205819. doi: 10.1371/journal.pone.0205819 (PMC6245515; doi:10.1371/journal.pone.0205819)
Supplement: S2 Table — (PDF) [file pone.0205819.s007.pdf]

Table S2: The comparison of residue-contact (top L/5 and L/2) among metapsicov , NeBcon and plmDCA.

PDB ID	Top L/5			Top L/2		
	Metapsicov [5]	NeBcon [6]	plmDCA	Metapsicov [5]	NeBcon [6]	plmDCA
1DMB	0.068	0.068	0.041	0.054	0.043	0.059
1E6K	0.231	0.269	0.154	0.231	0.185	0.123
1F21	0.000	0.000	0.000	0.000	0.056	0.014
1N0S	0.029	0.000	0.029	0.070	0.000	0.081
1R9H	0.000	0.000	0.000	0.000	0.000	0.000
1RQM	0.238	0.286	0.238	0.231	0.250	0.154
1SVN	0.057	0.057	0.075	0.030	0.030	0.052
1TVG	0.000	0.000	0.000	0.000	0.000	0.000
1ZGG	0.167	0.267	0.167	0.133	0.173	0.120
2JSZ	0.061	0.091	0.121	0.108	0.084	0.120
2K1S	0.107	0.071	0.107	0.043	0.086	0.057
2KPT	0.087	0.043	0.000	0.140	0.088	0.018
2KSY	0.000	0.000	0.000	0.000	0.000	0.000
2LCG	0.038	0.038	0.000	0.015	0.015	0.015
2LCI	0.115	0.115	0.192	0.179	0.209	0.134
2LTM	0.000	0.000	0.000	0.000	0.038	0.000
3MER	0.088	0.029	0.088	0.081	0.070	0.081
5P21	0.000	0.000	0.030	0.024	0.060	0.012
