# Supplementary material for: De novo protein structure prediction using ultra-fast molecular dynamics simulation
Source: PLoS One. 2018 Nov 20;13(11):e0205819. doi: 10.1371/journal.pone.0205819 (PMC6245515; doi:10.1371/journal.pone.0205819)
Supplement: S3 Table — (PDF) [file pone.0205819.s008.pdf]

Table S3: Comparison (whole length) between EVfold and NiDelta on the benchmark proteins.

PDB ID	EVfold		NiDelta	
	RMSD (Å)	TM score	RMSD(Å)	TM score
1DMB	12.627	0.3857	6.49	0.64
1E6K	4.017	0.6477	4.21	0.64
1F21	4.533	0.6210	5.47	0.56
1N0S	11.632	0.3315	5.02	0.59
1R9H	7.671	0.5929	3.34	0.68
1RQM	9.070	0.3506	2.12	0.80
1SVN	7.977	0.7302	5.17	0.69
1TVG	14.305	0.2604	4.77	0.63
1ZGG	3.672	0.7065	2.52	0.82
2JSZ	17.073	0.2987	2.38	0.83
2K1S	13.173	0.3384	3.11	0.76
2KPT	12.516	0.2849	2.19	0.80
2KSY	10.585	0.4033	5.24	0.65
2LCG	3.910	0.7298	2.51	0.79
2LTM	12.622	0.2633	3.42	0.69
3MER	14.744	0.4071	4.99	0.63
5P21	4.736	0.6890	2.98	0.77
2LCI*	–	–	4.08	0.68

*EVfold web-server cannot predict the structure of the protein.
